# High Blood Pressure in Obese and Nonobese Japanese Children: Blood Pressure Measurement is Necessary Even in Nonobese Japanese Children

**DOI:** 10.2188/jea.JE20090207

**Published:** 2010-09-05

**Authors:** Takako Shirasawa, Naoki Shimada, Hirotaka Ochiai, Tadahiro Ohtsu, Hiromi Hoshino, Rimei Nishimura, Aya Morimoto, Naoko Tajima, Akatsuki Kokaze

**Affiliations:** 1 Department of Public Health, Showa University School of Medicine, Tokyo, Japan; 2 Division of Diabetes, Metabolism and Endocrinology, Department of Internal Medicine, Jikei University School of Medicine, Tokyo, Japan

**Keywords:** high blood pressure, children, BMI, hypertensive family history

## Abstract

**Background:**

Although the prevalences of obesity and hypertension (HT) are increasing in children, there have been few epidemiological studies of HT in Japanese children. We evaluated the prevalences of HT and high-normal blood pressure (HNBP), and examined the relationship between blood pressure (BP) and body mass index (BMI), in Japanese children.

**Methods:**

The subjects of this study were 2420 children living in the town of Ina, Saitama Prefecture, Japan during the period from 2006 through 2008. Body height, weight, and BP were measured. HT and HNBP were defined according to the HT criteria for Japanese children. Children with HNBP or HT were defined as having high blood pressure (HBP).

**Results:**

The prevalences of HBP were 15.9% and 15.8% in fourth-grade boys and girls, respectively, and 11.1% and 10.8% in seventh-grade boys and girls, respectively. Irrespective of sex or grade level, a higher BMI was associated with a higher prevalence of HBP (*P* < 0.001). When compared with the <50th percentile BMI category, the crude odds ratios (ORs) were statistically significant for the 75th to 84th percentile category in fourth-grade boys (OR: 4.54, 95% CI: 2.36–8.76), the ≥95th percentile in fourth-grade girls (13.29, 5.93–29.77), the 85th to 94th percentile (3.16, 1.46–6.84) in seventh-grade boys, and the ≥95th percentile (7.96, 3.18–19.93) in seventh-grade girls.

**Conclusions:**

BMI was associated with HBP in Japanese school children. In addition, some children in the lower BMI categories also had HBP.

## INTRODUCTION

In recent years, the rising obesity epidemic has been paralleled by a similar epidemic in hypertension (HT).^1^ The prevalence of childhood obesity increased from 5% to 11% in the United States from the 1960s to the 1990s.^2^ An analysis of nationwide surveys from 1963 to 2000 found that childhood HT has been increasing in US children since the late 1980s.^3^

In Japan, there has been little opportunity to measure blood pressure (BP) among children, because BP measurement was not included in annual health check-ups of elementary and junior high school. Therefore, there have been few epidemiological studies on BP in Japanese children.^4^^,^^5^ Thus, the objective of the present study was to evaluate the prevalences of HT and high-normal BP (HNBP), and to examine the relationship between BP and body mass index (BMI), in Japanese children.

## METHODS

### Subjects

As part of its community health services, the town of Ina in Saitama Prefecture, Japan, provides annual health check-ups to prevent childhood lifestyle-related diseases.^6^^–^^8^ The subjects of this study were 2420 children who were either elementary school fourth graders (age 9–10 years) or first-year junior high school students (seventh graders, age 12–13 years) during the period from 2006 to 2008. Written, informed consent was obtained from each subject’s parent or guardian, and the study protocol was approved by the Medical Ethics Committee of Showa University School of Medicine (Approval No. 127).

### Data collection

The following information was collected for each child from his or her parent or guardian by using a self-administered questionnaire: age, sex, and past history of HT in the subject’s father and mother (ie, family history of HT). In the annual school health examination, all subjects were asked to remove their shoes and socks, after which their height and weight were measured in increments of 0.1 cm and 0.1 kg, respectively, while they were wearing light clothing. BMI was calculated as body weight (kg) divided by the square of height (m).

### Blood pressure measurement

After instructing each subject to sit and rest, a health check-up nurse measured BP in the right upper arm using a mercury manometer and a stethoscope. The size of the cuff was 9 cm for the fourth graders and 12 cm for the seventh graders.^9^ For fourth graders for whom the 9 cm cuff was too small, the 12 cm cuff was used. When systolic blood pressure (SBP) was ≥120 mm Hg or diastolic blood pressure (DBP) was ≥70 mm Hg, BP was measured 3 times, and the value at the third measurement was recorded.

### Determination of HT and HNBP

According to the HT criteria for Japanese children (Japanese Society of Hypertension Guidelines Subcommittee for the Management of Hypertension; JSH2004),^9^ HT and HNBP were defined as follows. In fourth graders, an SBP ≥135 mm Hg or DBP ≥80 mm Hg was defined as HT. In seventh graders, an SBP ≥140 mm Hg or DBP ≥85 mm Hg was defined as HT in boys, while an SBP ≥135 mm Hg or DBP ≥80 mm Hg was defined as HT in girls. A BP between normal BP (NBP) and HT was regarded as HNBP (fourth graders: 125 ≤ SBP < 135 or 70 ≤ DBP < 80, seventh-grade boys: 130 ≤ SBP < 140 or 70 ≤ DBP < 85, seventh-grade girls: 125 ≤ SBP < 135 or 70 ≤ DBP < 80). Children with either HNBP or HT were considered to have high blood pressure (HBP).

### Statistical analysis

The Mann–Whitney U-test was used to compare characteristics between boys and girls after the normality of distribution was tested for each variable. In the stratified analysis by sex and grade level, the relationship between HBP and BMI was investigated using the chi-square test and a logistic regression model. In the analysis of the association of HBP with BMI and family history of HT, BMI was analyzed as a categorical variables (<50th, 50th to 74th, 75th to 84th, 85th to 94th, and ≥95th percentile). A *P* value <0.05 was considered statistically significant. Data were analyzed using SPSS statistical analysis software package (16.0J).

## RESULTS

Among 2420 subjects, 35 were excluded from the analysis because of refusal to participate or school absence. Thus, data from a total of 2385 were analyzed. The rates of participation for the fourth and seventh graders were 98.7% (1297 of 1314 children) and 98.4% (1088 of 1106 children), respectively.

Table 1 shows the characteristics of the participants. Of the 2385 children, 1297 were fourth graders (661 boys and 636 girls) and 1088 were seventh graders (566 boys and 522 girls), with median ages of 9.0 years and 12.0 years, respectively. Median BMIs were 16.5 and 16.2 for fourth-grade boys and girls, respectively, and 17.9 and 18.3 for seventh-grade boys and girls. Median SBPs for fourth-grade boys and girls were 108 and 107 mm Hg, respectively, while those for seventh-grade boys and girls were 110 and 105 mm Hg; in both grades, SBP was significantly higher in boys than in girls (fourth graders: *P* = 0.009; seventh graders: *P* < 0.001). DBP was significantly higher in fourth-grade boys than in fourth-grade girls (*P* = 0.026), but no significant sex difference in DBP was seen in seventh graders. The proportions of children with a family history of HT were 3.6% and 4.2% for fourth-grade boys and girls, respectively, while those for seventh-grade boys and girls were 5.8% and 6.1%.

**Table 1.** Characteristics of subjects

	Fourth graders (age 9–10 years)			Seventh graders (age 12–13 years)		
		
	Boys (*n* = 661)	Girls (*n* = 636)	*P*-value	Boys (*n* = 566)	Girls (*n* = 522)	*P*-value
Age (years)	9.0 (9.2)	9.0 (9.3)	0.082	12.0 (12.3)	12.0 (12.3)	0.433
Height (cm)	134.6	133.8	0.198	154.5	152.7	0.001
Weight (kg)	30.0	29.2	0.006	42.9	43.3	0.974
BMI (kg/m^2^)	16.5	16.2	0.004	17.9	18.3	0.005
SBP (mm Hg)	108.0 (109.3)	107.0 (107.7)	0.009	110.0 (109.8)	105.0 (106.7)	<0.001
DBP (mm Hg)	60.0 (58.3)	58.0 (57.4)	0.026	56.0 (56.6)	56.0 (57.3)	0.323
Family history of HT (%)	3.6	4.2	0.669	5.8	6.1	0.898

Data are expressed as a median (mean) or percentage (%).

Mann–Whitney U-test or the chi-square test.

BMI, body mass index; SBP, systolic blood pressure; DBP, diastolic blood pressure; HT, hypertension.

The prevalences of HBP in fourth-grade boys and girls were 15.9% and 15.8%, respectively, and those for seventh-grade boys and girls were 11.1% and 10.8% (Table 2). In both grades, there was no significant difference between boys and girls in the prevalence of HBP; however, the prevalence of HBP was significantly higher among fourth graders than among seventh graders (boys: *P* = 0.016, girls: *P* = 0.015; data not shown).

**Table 2.** Prevalence of high blood pressure by sex

	Boys				Girls				*P*-value
		
	*n*	HBP	HNBP	HT	*n*	HBP	HNBP	HT	
Fourth graders (age 9–10 years)									
SBP	661	53 (8.0)	51 (7.7)	2 (0.3)	636	46 (7.2)	42 (6.6)	4 (0.6)	0.603
DBP	661	72 (10.9)	68 (10.3)	4 (0.6)	636	73 (11.5)	61 (9.6)	12 (1.9)	0.792
SBP and/or DBP	661	105 (15.9)	100 (15.1)	5 (0.8)	636	100 (15.8)	85 (13.4)	15 (2.4)	0.939

Seventh graders (age 12–13 years)									
SBP	566	28 (4.9)	28 (4.9)	0 (0.0)	522	24 (4.6)	24 (4.6)	0 (0.0)	0.887
DBP	566	42 (7.4)	42 (7.4)	0 (0.0)	522	41 (7.9)	38 (7.3)	3 (0.6)	0.820
SBP and/or DBP	566	63 (11.1)	63 (11.1)	0 (0.0)	522	56 (10.8)	53 (10.2)	3 (0.6)	0.846

Data are expressed as number (%).

Blood pressure (BP) was assessed according to the HT criteria for Japanese children (JSH2004).

The chi-square test was used to compare the prevalence of high blood pressure (HBP) between boys and girls.

HBP includes HNBP and HT.

SBP, systolic blood pressure; DBP, diastolic blood pressure; HNBP, high-normal BP; HT, hypertension.

The associations of HBP with BMI and a family history of HT are shown in Table 3. A higher BMI category was associated with a higher prevalence of HBP regardless of sex or grade level (*P* < 0.001). Among fourth graders, there was no significant association between HBP and a family history of HT in either sex; however, among seventh-grade boys, the prevalence of HBP was significantly higher in those with a family history of HT than in those without such a history (*P* = 0.022). There was no significant difference among seventh-grade girls (*P* = 0.561).

**Table 3.** Associations of high blood pressure with BMI and family history of hypertension, by sex

	Fourth graders (age 9–10 years)						Seventh graders (age 12–13 years)					
		
	Boys (*n* = 661)			Girls (*n* = 636)			Boys (*n* = 566)			Girls (*n* = 522)		
				
	NBP	HBP	*P*-value	NBP	HBP	*P*-value	NBP	HBP	*P*-value	NBP	HBP	*P*-value
BMI percentile												
<50th	253 (90.7)	26 (9.3)	<0.001	288 (90.6)	30 (9.4)	<0.001	261 (92.2)	22 (7.8)	<0.001	242 (92.7)	19 (7.3)	<0.001
50–74th	189 (87.1)	28 (12.9)		128 (80.5)	31 (19.5)		130 (92.2)	11 (7.8)		117 (89.3)	14 (10.7)	
75–84th	45 (68.2)	21 (31.8)		53 (84.1)	10 (15.9)		49 (86.0)	8 (14.0)		47 (90.4)	5 (9.6)	
85–94th	49 (74.2)	17 (25.8)		54 (83.1)	11 (16.9)		54 (78.9)	12 (21.1)		44 (84.6)	8 (15.4)	
≥95th	20 (60.6)	13 (39.4)		13 (41.9)	18 (58.1)		18 (64.3)	10 (35.7)		16 (61.5)	10 (38.5)	

Family history of hypertension												
No	537 (84.3)	100 (15.7)	0.566	514 (84.4)	95 (15.6)	0.597	478 (89.7)	55 (10.3)	0.022	436 (89.0)	54 (11.0)	0.561
Yes	19 (79.2)	5 (20.8)		22 (81.5)	5 (18.5)		25 (75.8)	8 (24.2)		30 (93.8)	2 (6.2)	

Data are expressed as number (%).

The chi-square test was used to investigate the associations of HBP with BMI and a family history of hypertension.

NBP, normal blood pressure; HBP, high blood pressure; BMI, body mass index.

Logistic regression analysis was conducted using HBP as an objective variable to calculate the odds ratio (ORs) of HBP and their 95% confidence intervals (95% CIs) (Table 4). With the <50th percentile BMI category as the reference, the crude OR was statistically significant for the 75th to 84th percentile category of fourth-grade boys (OR: 4.54, 95% CI: 2.36–8.76), the ≥95th percentile of fourth-grade girls (13.29, 5.93–29.77), the 85th to 94th percentile (3.16, 1.46–6.84) of seventh-grade boys, and the ≥95th percentile (7.96, 3.18–19.93) of seventh-grade girls. The adjusted ORs were similar to the crude ORs.

**Table 4.** Odds ratios and 95% confidence intervals of high blood pressure by BMI and sex

	Boys					Girls				
		
	*n*	Crude		Adjusted^a^		*n*	Crude		Adjusted^a^	
				
		OR	95% CI	OR	95% CI		OR	95% CI	OR	95% CI
Fourth graders (age 9–10 years)										
BMI percentile										
<50th	279	1		1		318	1		1	
50–74th	217	1.44	(0.82–2.54)	1.45	(0.82–2.56)	159	2.33	(1.35–4.00)	2.32	(1.34–3.99)
75–84th	66	4.54	(2.36–8.76)	4.52	(2.34–8.72)	63	1.81	(0.84–3.93)	1.81	(0.84–3.93)
85–94th	66	3.38	(1.70–6.69)	3.43	(1.73–6.82)	65	1.96	(0.92–4.14)	1.95	(0.92–4.13)
≥95th	33	6.33	(2.82–14.17)	6.30	(2.81–14.12)	31	13.29	(5.93–29.77)	13.32	(5.95–29.84)
		(*P* for trend <0.001)		(*P* for trend <0.001)			(*P* for trend <0.001)		(*P* for trend <0.001)	

Seventh graders (age 12–13 years)										
BMI percentile										
<50th	283	1		1		261	1		1	
50–74th	141	1.00	(0.47–2.13)	1.02	(0.48–2.16)	131	1.52	(0.74–3.15)	1.50	(0.72–3.09)
75–84th	57	1.94	(0.82–4.60)	1.83	(0.76–4.37)	52	1.36	(0.48–3.81)	1.33	(0.47–3.74)
85–94th	57	3.16	(1.46–6.84)	3.00	(1.38–6.54)	52	2.32	(0.96–5.62)	2.27	(0.94–5.53)
≥95th	28	6.59	(2.72–16.00)	6.32	(2.59–15.45)	26	7.96	(3.18–19.93)	7.83	(3.12–19.63)
		(*P* for trend <0.001)		(*P* for trend <0.001)			(*P* for trend <0.001)		(*P* for trend <0.001)	

^a^Adjusted for family history of hypertension.

BMI, body mass index; OR, odds ratio; 95% CI, 95% confidence interval.

## DISCUSSION

In this study, the prevalences of HBP in fourth and seventh graders were approximately 16% and 11%, indicating that there was an approximately 5% difference in HBP prevalence between fourth and seventh graders. Previous studies have shown that BP in childhood was not influenced by age,^10^ but that physiological and/or endocrinological factors do affect BP.^11^^,^^12^ Therefore, future study will be necessary to examine this question from the perspectives of physiology and endocrinology.

A higher BMI was associated with an increased prevalence of HBP, as was previously reported.^13^^–^^15^ In particular, the adjusted OR for fourth-grade boys reached statistical significance at the 75th to 84th percentile BMI category, which would be categorized as a healthy weight if the weight status categories of the Centers for Disease Control and Prevention (CDC)^16^ were applied. This suggests that BP measurement is necessary even for children who are not overweight or obese.

In this study, a statistically significant association between HBP and a family history of HT was observed in seventh-grade boys. This result was consistent with that of previous studies, which showed that BP in children was related to a family history of HT.^14^^,^^17^^,^^18^ Furthermore, a parental history of HT before age 60 was reported to be related to HT in their children.^19^ Therefore, we reanalyzed our data after excluding subjects with hypertensive fathers or mothers aged 60 or older. However, the findings were similar to those shown in Tables 3 and 4 (data not shown).

The mean SBP and DBP of the children in this study were similar to those noted in previous studies.^4^^,^^20^^–^^22^ However, in our study, when SBP was ≥120 mm Hg or DBP was ≥70 mm Hg, BP was measured 3 times and the third measurement was recorded. Therefore, some children who would have been classified as having HBP on the basis of the first measurement were categorized as having NBP if they did not meet the criteria of HBP on the third measurement. Thus, the prevalence of HBP in this study might have been underestimated, because previous research found that the third measurement of BP was the lowest of 3 measurements.^23^

A couple of studies found that children with HBP were likely to develop essential HT when they reach adulthood.^24^^,^^25^ Therefore, it is important to screen BP as early as possible and to begin health education at the first appearance of HNBP. However, BP can be affected by several factors. Some children are unfamiliar with BP measurement and their anxiety might affect their BP. Thus, it is important to measure BP periodically, so that children are familiar with the measurement technique and the effect of anxiety is minimized. Additionally, Kikuchi et al^26^ reported that BP measurement is an excellent part of health education, because BP measurement is noninvasive, inexpensive, and requires only a short period of time. Accordingly, lifestyle and health guidance should be provided not only to hypertensive children, but also to those with HNBP.

A limitation of the present study was its cross-sectional design. Therefore, the direction of causality cannot be determined with respect to BMI and HBP. In the future, longitudinal research will be necessary to address this question. A family history of HT, which has been reported to be associated with BP,^14^^,^^18^ was included as a potential confounder in the logistic regression model. However, because the possibility of residual confounding factors cannot be denied, it is necessary to conduct another study that adjusts for other potential confounding factors, such as diet and exercise. In addition, the subjects in this study were children from only 1 town in Japan. Therefore, it is difficult to generalize these results to all Japanese children.

In conclusion, our data suggest that higher BMI is associated with a higher prevalence of HBP. However, children in the lower BMI categories also had HBP. Therefore, BP measurement is necessary even for these children. To realize this hope of BP measurement for all children, it is necessary to add BP measurement as a required test in preschool and school health check-ups, so that BP measurement is available for both clinical and preventive medicine. This should facilitate early lifestyle interventions that may prevent HBP, resulting in a contribution to primary prevention for lifestyle-related diseases, including HT.
